# Abundance, distribution, and growth characteristics of three keystone *Vachellia* trees in Gebel Elba National Park, south-eastern Egypt

**DOI:** 10.1038/s41598-020-79542-1

**Published:** 2021-01-14

**Authors:** Ahmed M. Abbas, Mohammed Al-Kahtani, Stephen J. Novak, Wagdi Saber Soliman

**Affiliations:** 1 Department of Biology, College of Science, King Khalid University, Abha, 61413 Saudi Arabia; 2 Department of Botany and Microbiology, Faculty of Science, South Valley University, Qena, 83523 Egypt; 3 Department of Biological Sciences, Boise State University, Boise, ID 83725 USA; 4 Department of Horticulture, Faculty of Agriculture and Natural Resources, Aswan University, Aswan, 81528 Egypt

**Keywords:** Ecology, Plant sciences, Environmental sciences

## Abstract

This study was conducted to evaluate the abundance and distribution pattern of three keystone *Vachellia* taxa in wadi Khoda and wadi Rahaba, Gebel Elba National Park, a protected area in south-eastern Egypt. These taxa included *Vachellia tortilis* subsp. *tortilis*, *Vachellia tortilis* subsp. *raddiana*, and *Vachellia ehrenbergiana*. In wadi Khoda, only two of these taxa were detected (*V*. *tortilis* subsp. *raddiana* and *V*. *tortilis* subsp. *tortilis*), while all three taxa were encountered in wadi Rahaba. The density of trees in wadi Khoda was 34.3 plant ha^−1^ compared to 26.3 plant ha^−1^ in wadi Rahaba. *Vachellia tortilis* subsp. *raddiana* was the most frequently observed tree, with an average of 47.93% and 68.25% in wadi Rahaba and wadi Khoda, respectively. Crown depth and tree height were mainly associated with elevation, indicating that elevation plays a key role in the growth of these *Vachellia* taxa. Our results suggest that human activities have had limited effect on the distribution of these three keystone taxa. Thus, management in Gebel Elba National Park should continue to limit the impact of human activities on these three keystone species.

## Introduction

The keystone species concept is an important aspect of population ecology, community ecology, and conservation biology^1,2^, and its application is likely to be critical with ongoing climate change^3^. Keystone species can be identified because they have a larger effect on communities and ecosystems than would be predicted based on their abundance or dominance. Loss of keystone species within communities and ecosystems is likely to result in secondary extinction events, and in extreme cases these events can lead to community and ecosystem collapse^4^. The critical importance of keystone species is derived from the wide range of biotic interactions they engage in with other community members (predation, competition, herbivory, mutualism, facilitation, etc.) and their influence on abiotic environmental conditions^2^. Keystone species have been described in a range of ecosystems (e.g., marine, fresh water, terrestrial, etc.) and have included a variety of taxa (e.g., fungi, animals, and plants)^1,3,5^.

Plant communities consisting of isolated or scattered trees occur across the globe, and such trees have been described as keystone species, or “keystone structures”^6^. This certainly applies to trees and shrubs that are members of plant communities in arid and semi-arid habitat^7^. Many members of *Acacia s*.*l*. (Fabaceae: Mimosoideae^8^), which are broadly distributed around the world, are considered keystone species within the communities they reside. For example, they are considered keystone species in parts of Australia^9^, Pakistan^10^, the Kalahari Desert, Botswana^11^, Tunisia^12–14^, the Sinai Desert, Egypt^15,16^, and south-eastern Egypt^16,17^. As pointed out by Abdallah et al.^12^, isolated trees in arid habitats, including *Vachellia* species., have several characteristics that contribute to their keystone status: (1) shade from their canopies prevents extreme temperature fluctuations, increases soil moisture levels, and provides shelter for wildlife, (2) they improve soil conditions through biological nitrogen fixation and litter fall by increasing soil nitrogen content, organic carbon, and water-holding capacity, (3) they increase plant and animal biodiversity as a consequence of characteristics one and two, (4) they provide a source of food for wildlife, and (5) they provide a source of fuel, fodder, and medicines for local people and their domesticated animals. Because of their critical importance, a full characterization of keystone species and the roles they play within communities and ecosystems is urgently needed; especially as they are adversely impacted by various human activities.

The Gebel Elba mountain range is an extension of the Afromontane “biodiversity hotspot” and is at the northern limit of the Eritreo-Arabian province and the Sahel regional transition zone^18^. The relatively high abundance of moisture of this mountain range leads to higher plant biodiversity than reported elsewhere in Egypt, it consists of 458 species, which constitutes approximately 21% of the Egyptian flora^19,20^. According to the plant checklist provided by Boulos^21^, the flora of Egypt consists of 2100 taxa belonging to 755 genera and 129 families; including 45 genera and 228 taxa in the Fabaceae. Gebel Elba is one of the seven main phytogeographical regions in Egypt^21^. Additionally, the region’s tree and shrub species diversity is higher than in any other regions in Egypt^19^, with some Sahelian woody elements restricted to the Gebel Elba region and not reported elsewhere in Egypt. Of the 10 *Vachellia* (synonym: *Acacia*^8^) species reported in Egypt, seven are known to occur in the Gebel Elba region, with *Vachellia asak* (synonym: *Acacia asak*) and *Vachellia oerfota* subsp. *oerfota* (synonym: *Acacia oerfota* subsp. *oerfota*) restricted to this region.

An analysis of the plant communities of wadi Yahmib and three of its tributaries, on the north-western slopes of Gebel Elba, revealed the presence of seven plant communities, with these communities being arrayed across an elevational (environmental) gradient^17^. The *Vachellia tortilis* subsp. *tortilis* (synonym: *Acacia tortilis* subsp. *tortilis*) community was the main vegetation type on Gebel Elba. This community type occurred commonly in the water channels of wadis and gravel terraces from low to mid elevations (130–383 m), and the species was a member of all of the other six communities in the study area^17^. In addition, *Vachellia tortilis* subsp. *raddiana* (synonym: *Acacia tortilis* subsp. *raddiana*) was an overstory co-dominant species in another community on Gebel Elba. Finally, a third acacia species, *Vachellia etbaica* (synonym: *Acacia etbaica*), was also detected in this study.

Within arid and semi-arid ecosystems across north Africa and the Arabian Peninsula, plant ecologists have focused their attention on describing the vegetation of wadis that drain to the Red Sea, with these studies focusing on keystone *Vachellia* species^12–17,22,23^. The present study aimed to contribute to this body of knowledge by determining the distribution, abundance, and describing the growth characteristics of three *Vachellia* tree taxa in wadi Khoda and wadi Rahaba, in Gebel Elba National Park, south-eastern Egypt. These data will allow us to provide detailed descriptions of the characteristics of these three taxa. This study is essential at this moment because these tree taxa are keystone species within these ecosystems, and their presence and conservation are likely to be threatened by human activities and ongoing climate change.

## Materials and methods

### Study sites

**Figure 1:**
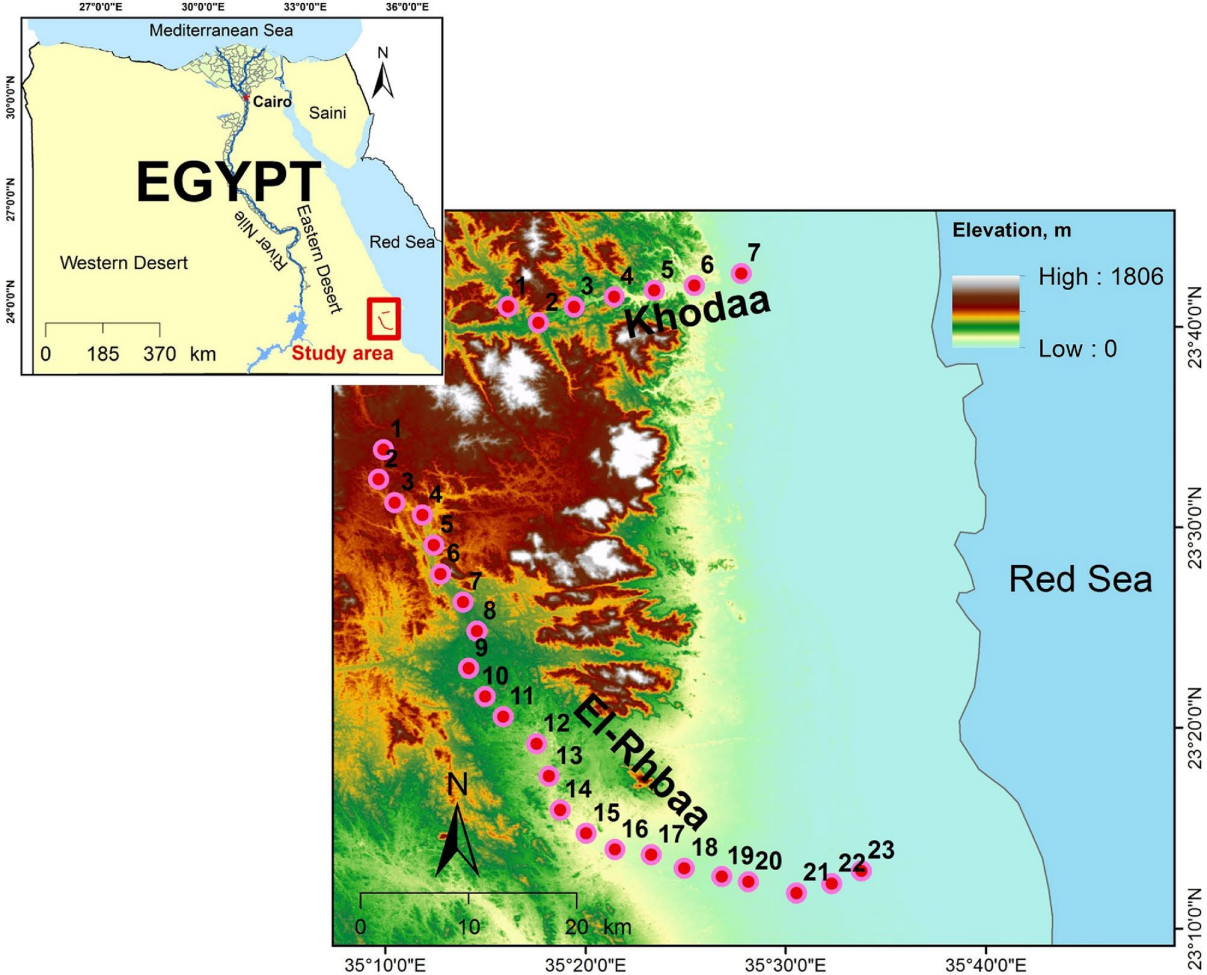
Map of the study region showing the location of the macroplots used to sample the three *Vachellia* tree taxa. Twenty-three macroplots were analyzed in wadi El Rahaba and seven macroplots were analyzed in wadi Khoda (*n* = 7), Gebel Elba National Park, south-eastern Egypt. Macroplots in each wadi are indicated numerically (ArcGIS 10.4.1).

The Gebel Elba National Park (35° 00′ E–37° 00′ E, 22° 00′ N–23° 50′ N) is located in the southeastern corner of Egypt and comprises an area of approximately 35,600 km^2^ (Fig. 1). The boundaries of this protected area extend more than 50 km north of Shalatein, Egypt, eastward to the Red Sea, south to the border with Sudan, and westward into the Eastern Desert^24^.

**Figure 2:**
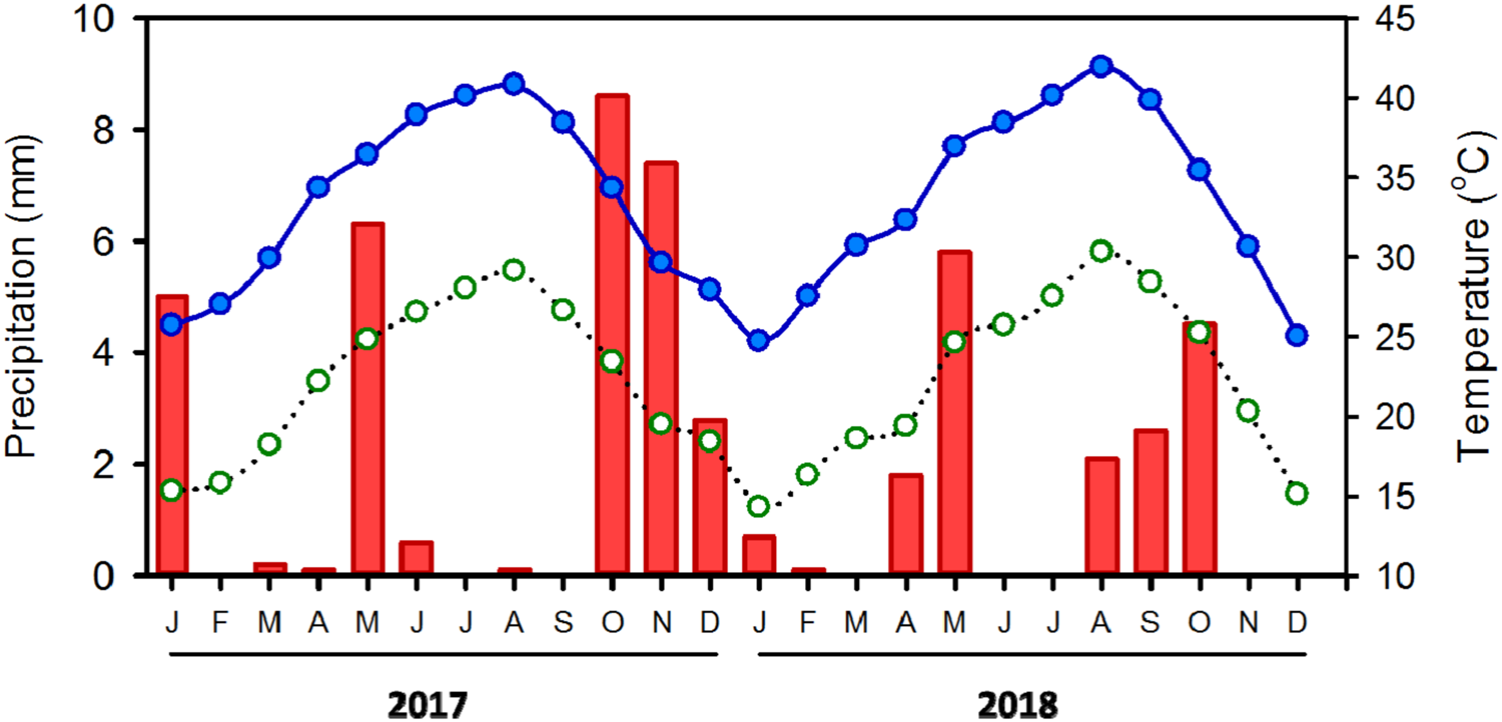
Monthly rainfall (mm; bars) and maximum (solid black circle) and minimum (open circle) temperature (°C) at wadi Rahaba and wadi Khoda, Gebel Elba National Park, south-eastern Egypt from January 2017 to December 2018.

This study was undertaken in 2017 and 2018. Over these two years, the average daily air temperature ranged between 25 and 29 °C, the maximum air temperatures were 40.8 °C in 2017 and 41.9 °C in 2018, the minimum air temperatures were 15.3 °C in 2017 and 14.3 °C in 2018. Annual precipitation was 31.1 mm in 2017 and 17.6 mm in 2018 (Fig. 2). Mean daily relative humidity ranged between 39 and 45% in 2017 and 38 and 44% in 2018, with a range of 28% to 61% relative humidity across the two years.

### Data collection

**Figure 3:**
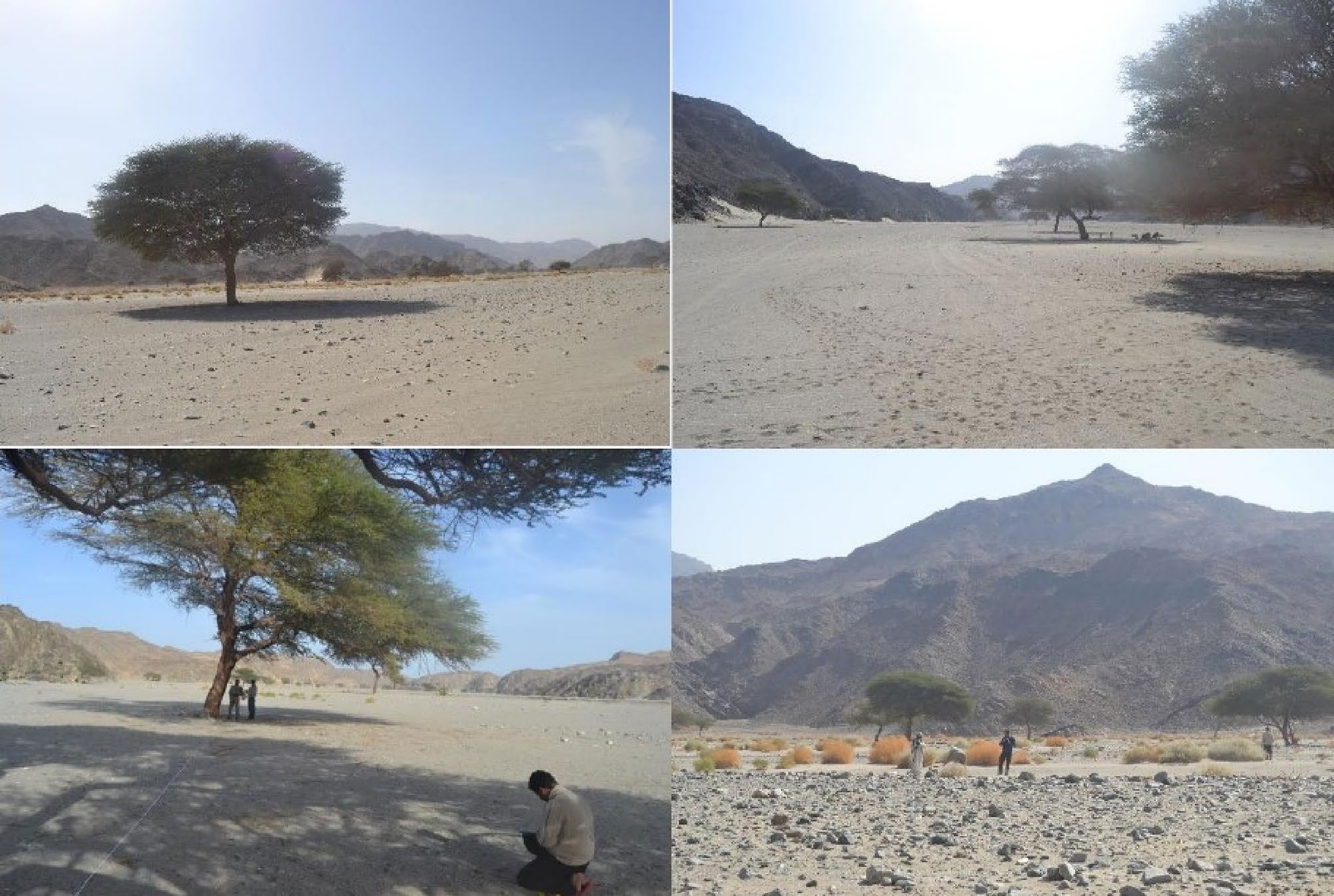
Photos showing two of the *Vachellia* tree taxa analyzed at the two wadis in Gebel Elba National Park, south-eastern Egypt. The two photos on the left side of the figure were taken at wadi Khoda and the two photos on the right side were taken at wadi Rahaba. *Vachellia tortilis* subsp. *tortilis *and *V*. *tortilis* subsp. *raddiana *are shown in these photos, but *V*. *ehrenbergiana *does not appear. The Gebel Elba mountain range is in the background of the bottom-right photo.

**Figure 4:**
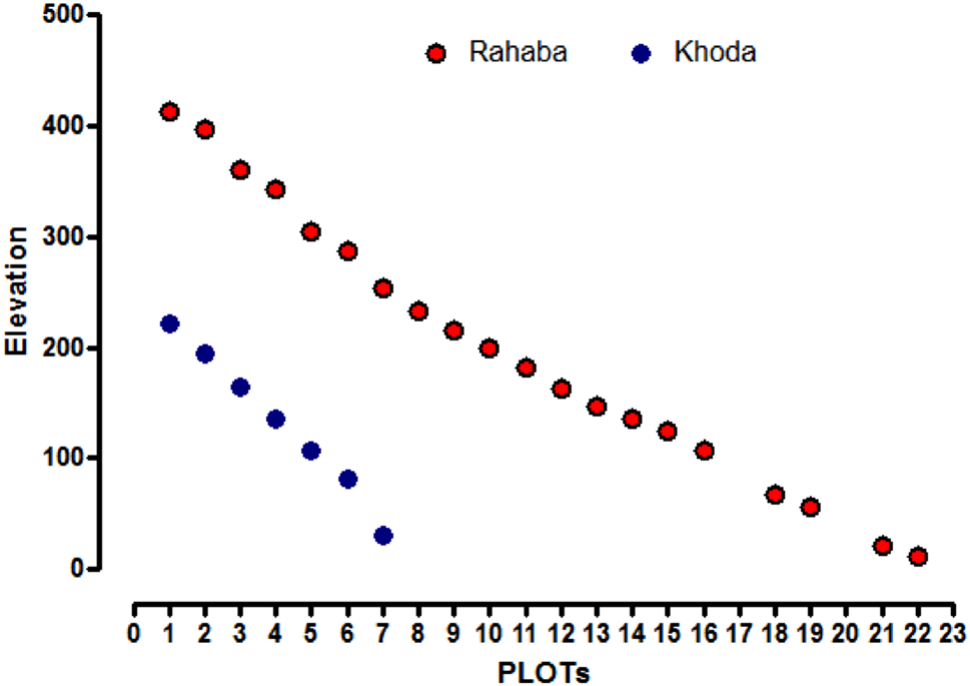
The elevation (m) of the macroplots in which *Vachellia* tree taxa were sampled. Red symbols indicate the elevation of the macroplots analyzed in wadi Rahaba and blue symbols indicate the elevation of the macroplots analyzed in wadi Khoda, Gebel Elba National Park, south-eastern Egypt.

Three *Vachellia* tree taxa; *Vachellia tortilis* subsp. *tortilis*, *Vachellia tortilis* subsp. *raddiana*, and *Vachellia ehrenbergiana* (synonym: *Acacia ehrenbergiana*) were the focus of this study because they are the only tree taxa present at the two study sites (there was no other woody vegetation). During each year of the study, a transect was established along the length of wadi Khoda and wadi Rahaba, in Gebel Elba National Park. We chose these two areas for this study because these three *Vachellia* tree taxa are common in these wadis (Fig. 3). The transect in wadi Rahaba had a north to south-east orientation and the transect in wadi Khoda had a west to east orientation. The direction of the transect in each wadi was determined by the direction of the wadi’s drainage system. A total of 30 macroplots (200 m × 100 m) were established in the two wadis (Rahaba, n = 23; Khoda, n = 7), with distances of approximately 3 km between each macroplot (Figs. 1, 4).

For each macroplot, all tree taxa were identified according to Boulos^19^. Because this study was conducted during a period of drought, saplings and seedlings were only rarely observed in the two wadis. Consequently, we only encountered mature trees with a DBH > 10 cm in the macroplots we sampled. The tree characteristic parameters we measured included crown depth, crown diameter, crown volume, tree height, Diameter at Breast Height, DBH (tree diameter), tree radius, and the basal area of each tree^25^. The crown-to height ratio (C/H ratio) was calculated by dividing the crown volume by tree height. Crown diameter was measured by projecting several edges of the crown to the ground and measuring the length along an axis that extended from crown edge to crown edge. The crown diameter can be used to estimate the area of a tree’s crown (which is used in the crown surface area and volume calculations) by obtaining the average of two axes^26^. In this study, the diameters of two axes perpendicular to each other (N–S and E–W) were measured and averaged. Crown depth was measured as the distance between the top of the tree and the base of the crown. The base of the crown was identified by the lowest complete whorl of branches or the lowest single branch that formed the canopy of a tree. Crown depth was expressed as the "crown length ratio", which is calculated as the crown length divided by the tree height. Crown volume (Cv) was estimated from crown diameter (D) and crown depth (L) using the equation described by West^26^:

1 $$ {\text{Cv}} = \pi {\text{D2L}}/{12} $$

The trees in each wadi were numbered sequentially, and their latitude/longitude coordinates were recorded using a 12XL Garmin GPS unit. The nearest-neighbor distance for each pair of trees was measured and recorded. As noted by Cottam and Curtis^27^, many of these nearest-neighbor distance values are duplicates, since paired neighbors which have each other as nearest neighbors make up a large portion of the total dataset.

To determine the distribution pattern of the three *Vachellia* tree taxa we detected in this study, we used the method of Clark and Evans^28^. Briefly, in a population of N individuals with known density (d) and distance from each nearest-neighbor pair (r), the mean observed distance is calculated as follows:

2 $$ {\text{r}}0 = \Sigma {\text{r}}/{\text{N}} $$

The mean distance that would be expected if members of a population were randomly distributed (re) is calculated as:

3 $$ {\text{re}} = {1}/{2}\surd {\text{d}} $$

The degree to which the observed distribution for the distance to the nearest neighbor approaches or departs from random expectation was expressed as the ratio (R) as follows:

4 $$ {\text{R}} = {\text{r}}0/{\text{re}} $$

According to Clark and Evans^28^ and Petrere^29^, R has a defined range: 0.0 < R < 2.1491. If R = 0.0, individuals are highly aggregated, when R = 2.1491, there is a completely uniform distribution pattern, and when R = 1.0, the distribution pattern of individuals is random.

### Statistical analysis

Parametric statistical tests were performed on key variables after checking for normality and equality of variance. One-way analysis of variance (ANOVA) was used to evaluate statistical differences among the parameters used to characterize the three *Vachellia* tree taxa at wadi Rahaba. With only two tree taxa detected at wadi Khoda, *t*-tests were used to analyze significance among the mean values of these parameters. All statistical analyses were carried out using JMP (ver 4. SAS Institute, Cary, NC, USA).

## Results

The elevation of macroplots were significantly different (*P* < 0.0001), and the elevation of macroplots increased with distance from the Red Sea (Fig. 4). For wadi Khoda, the macroplot with the lowest elevation was located 29 m above sea level and the macroplot at the highest elevation was 224 m. For wadi Rahada, the elevation of the macroplots ranged from 11 to 418 m above sea level.

### Tree abundance, distribution, and density

All three *Vachellia* tree taxa were found in wadi Rahaba, while only two of these taxa (*V. tortilis* subsp. *raddiana* and *V. tortilis* subsp. *tortilis*) were observed in wadi Khoda. In wadi Khoda, seven trees, on average, were found in each macroplot (with density of 34.3 trees ha^−1^); these seven individuals consisting of, on average, five *V. tortilis* subsp. *raddiana* trees and two *V. tortilis* subsp. *tortilis* trees. There was an average of 5.26 trees per plot in wadi Rahaba, with a density of 26.3 trees ha^−1^ (even though no trees were observed in three of the macroplots in wadi Rahaba). *Vachellia tortilis* subsp. *raddiana* was the most frequently detected *Vachellia* tree taxa detected in the two wadis we analyzed (Table 1), it represented 68.75% of the trees at wadi Khoda and 47.93% of the trees at wadi Rahaba. *Vachellia tortilis* subsp. *tortilis* was the second-most common tree at both study sites (42.15% of the trees at wadi Rahaba and 31.25% of the trees at wadi Khoda). Only twelve *V*. *ehrenbergiana* trees were observed within the wadi Rahaba microplots (9.92% of the trees at this wadi) (Table 1).

**Table 1:** Range in the number of *Vachellia* tree taxa, mean number of trees, density, and percentage of each taxa in the macroplots in wadi Khoda and wadi Rahaba, Gebel Elba National Park, south-eastern Egypt.

Location	Species	Range	Mean	Density	Percentage (%)
Wadi Rahaba	*V. ehrenbergiana*	0–4	0.52	2.61	9.92
	*V. raddiana*	0–8	2.52	12.61	47.93
	*V. tortilis*	0–14	2.22	11.09	42.15
Wadi Khoda	*A. raddiana*	0–8	4.71	23.57	68.75
	*A. tortilis*	1–4	2.14	10.71	31.25

### Variation in tree characteristic parameters

**Figure 5:**
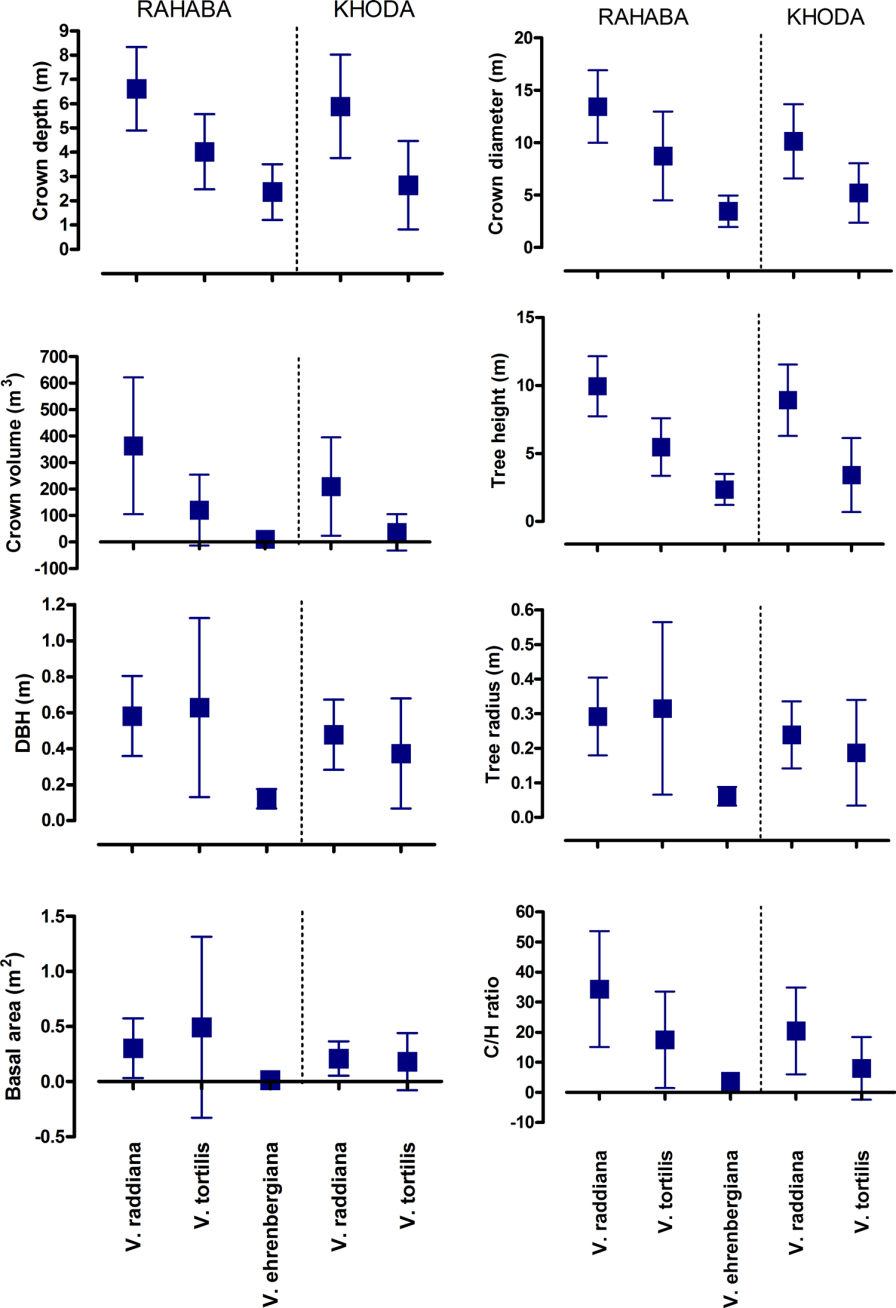
The tree characteristic parameters measured in this study. These parameters included crown depth (m), crown diameter (m), crown volume (m^3^), tree height (m), diameter at breast height (DBH) (m), tree radius (m), and crown volume/tree height ratio (C/H ratio) for the three *Vachellia* tree taxa in wadi Khoda and wadi Rahaba, Gebel Elba National Park, south-eastern Egypt. Statistical analyses of these data are provided in the text.

Values for these tree characteristic parameters varied among the three *Vachellia* tree taxa and between the two wadis where this study was conducted (Fig. 5). A comparison of *V. tortilis* subsp. *raddiana* and *A. tortilis* subsp. *tortilis* in the two wadis did not reveal significant differences between the two taxa for crown diameter and tree height (Fig. 5). However, these two *Vachellia* trees exhibited significantly higher values for crown depth, crown volume, DBH, tree radius, basal area, and C/H ratio in wadi Khoda.

In wadi Khoda, were only two *Vachellia* tree taxa were detected (*V. tortilis* subsp. *raddiana* and *V. tortilis* subsp. *tortilis*), *V. tortilis* subsp. *raddiana* had significantly higher values for crown depth (d.f. = 1; *t*-test 5.021; *P* < 0.0001), crown diameter (d.f. = 1; *t*-test 4.064; *P* = 0.0002), crown volume (d.f. = 1; *t*-test 3.419; *P* = 0.0013), tree height (d.f. = 1; t-test 6.402; *P* < 0.0001), and C/H ratio (d.f. = 1; *t*-test 2.868; *P* < 0.0063) compared to *V. tortilis* subsp. *tortilis*. There were no significant differences for DBH, tree radius, and basal area for these two species at wadi Khoda (Fig. 5).

In wadi Rahaba, three *Vachellia* tree taxa were encountered, and they had significant differences for the growth characteristics measured in this study. *Vachellia tortilis* subsp. *raddiana* had significantly greater values compared to *V. tortilis* subsp. *tortilis* and *V. ehrenbergiana* for crown depth (*F* = 40.00; *P* < 0.0001), crown diameter (*F* = 41.38; *P* < 0.0001), crown volume (*F* = 22.17; *P* < 0.0001), tree height (*F* = 71.22; *P* < 0.0001), and C/H ratio (*F* = 17.20; *P* < 0.0001). Whereas, *V. tortilis* subsp. *raddiana* and *V. ehrenbergiana* had greater values than *V. tortilis* subsp. *tortilis* for DBH (*F* = 11.97; *P* < 0.0001), tree radius (*F* = 11.97; *P* < 0.0001), and basal area (*F* = 4.48; *P* < 0.0085) (Fig. 5).

**Figure 6:**
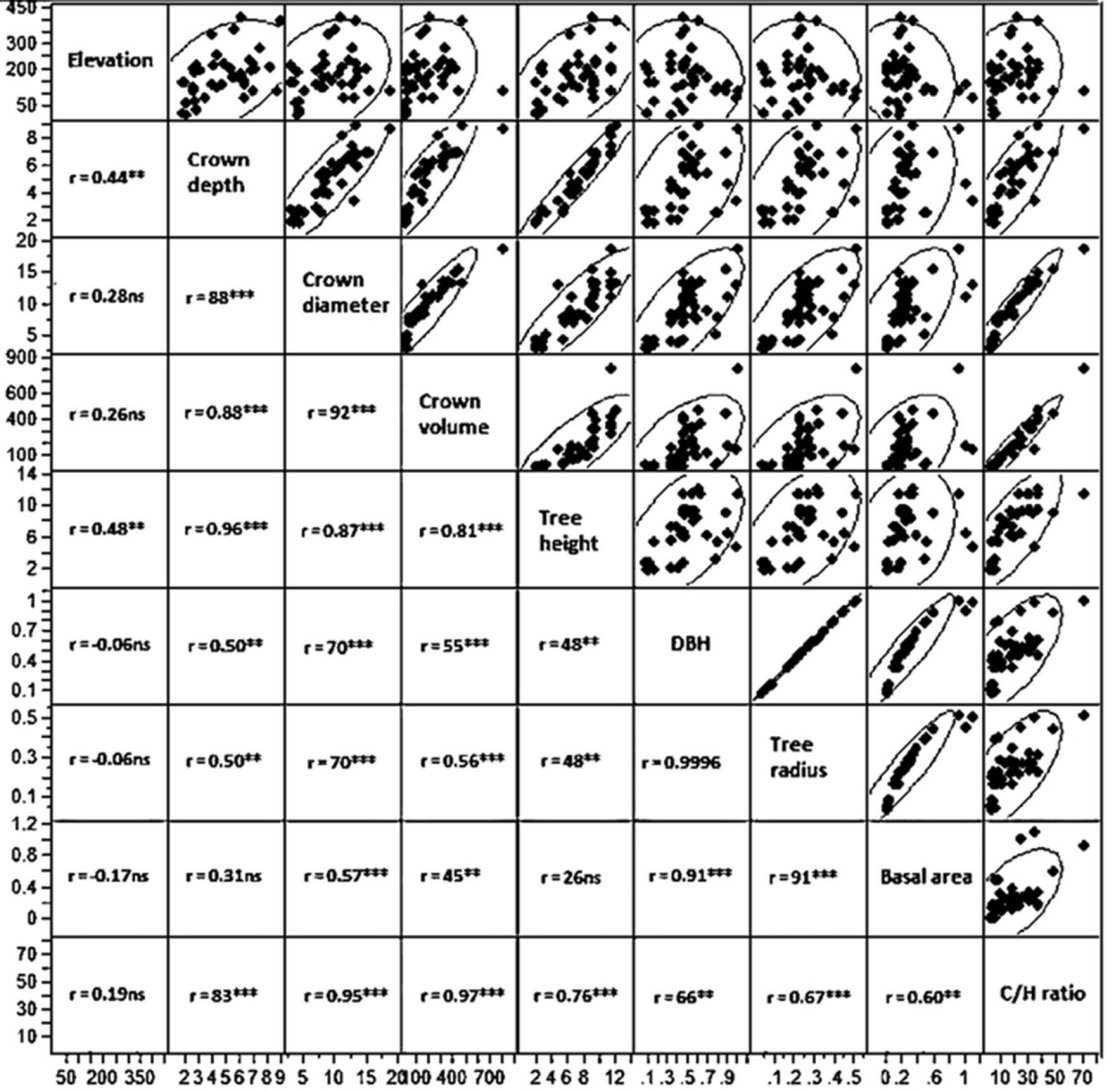
Scatter matrix of correlation coefficients of elevation and *Vachellia* tree characteristic parameters in wadi Khoda and wadi Rahaba, Gebel Elba National Park, southeastern Egypt.

Correlation coefficients of elevation with all tree characteristic parameters, as well as the correlation among tree characteristics were provided in Fig. 6. These results showed that crown depth and tree height are significantly associated with elevation (*r* = 0.44 and 0.48, respectively), while elevation was not significantly correlated with other tree characteristic parameters (see Fig. 6). Crown diameter, crown volume, DBH, and tress radius showed significantly positive correlation patterns. On the other hand, crown depth and tree height were significantly correlated with each other and other traits, but not basal area (Fig. 6).

### Mean observed nearest neighbor distance

The mean observed nearest neighbor distance (r_0_) was 60.1 ± 6.2 m in wadi Khoda macroplots, compared to 61.6 ± 6.8 m in wadi Rahaba plots (Table 2). The mean expected distance (r_e_) was shorter in the wadi Khoda macroplots (29.0 ± 2.7 m), compared with the value of r_e_ for trees in the wadi Rahaba macroplots (34.8 ± 2.6 m) (*t*-test, *P* < 0.05; Table 2). *R* values indicated that the distribution pattern of *Vachellia* tree taxa varied between random and completely uniform in both wadis. The *R* values were 1.6 and 1.7 in wadi Rahba and wadi Khoda, respectively (Table 2), and these values were significantly different (*t*-test, *P* < 0.05).

**Table 2:** Distribution pattern of the *Vachellia* tree taxa in wadi Khoda and wadi Rahaba, Gebel Elba National Park, south-eastern Egypt.

	Wadi Rahba	Wadi Khoda
Mean observed distance (m) [r_0_]	61.6 ± 6.8	60.1 ± 6.2
Mean expected distance (m) [r_e_]	34.8 ± 2.6	29.0 ± 2.7
R = r_0_/r_e_	1.6 ± 0.1	1.7 ± 0.2

Statistical analyses of these data are provided in the text.

## Discussion

Variation between and among plant populations arises due to differences in genetic diversity, heterogeneity of resource availability, competition, herbivory, and pathogen attack, all of which effect the growth rates of individuals in a population^30^. Topographic and edaphic factors, especially altitude, play a pivotal role in the distribution of plant communities. Understanding the relationships between vegetation cover and these environmental factors contributes to developing sustainable management strategies for such communities^31,32^.

Results of this study showed clear differences in populations of three *Vachellia* tree taxi in two wadis in Gebel Elba National Park, wadi Khoda and wadi Rahaba. Two *Vachellia* tree taxa were recorded in wadi Khoda (*V. tortilis* subsp. *raddiana* and *V. tortilis* subsp. *tortilis*), whereas all three taxa were detected at wadi Rahaba (*V. tortilis* subsp. *raddiana*, *V. tortilis* subsp. *tortilis*, and *V. ehrenbergiana*). Although *V. tortilis* subsp. *raddiana* was the most frequently encountered taxa in both wadis, *V. tortilis* subsp. *tortilis* predominated in low elevation macroplots, with its frequency decreasing with increasing elevation (i.e., distance from the Red Sea). These findings suggest that the distribution and abundance of the three *Vachellia* taxa in these wadis is likely influenced by topography and elevation. In addition, soil properties, especially soil salinity, may play a role in the distribution and abundance of these taxa. Thus, the findings of the current study concerning the diversity of the three *Vachellia* taxa documented in these two wadis and the distribution of these taxa along a broad elevation gradient are in general agreement with similar studies conducted in south-eastern Egypt^16,17,22^, and elsewhere across the distribution of *Vachellia* species^12–15,23^.

Previous studies have reported that the growth rates of individual trees were influenced by internal tree competitiveness as well as, limited resources, such as sunlight and water availability^33,34^. For instance, the mean mass of seven-year old *Acacia auriculiformis* trees decreased with increasing stand density^35^. Alternatively, a few studies have reported that tree seedlings planted at high density exhibited more rapid growth rates than seedlings planted at low density^36^. However, the positive-growth responses associated with high density was found to be a short-lived phenomenon.

Henskens et al*.*^37^ reported that mean tree mass (and other tree growth parameters) increased with decreasing stand density. This occurred due the following factors: (1) increased competition for sunlight among individuals in high-density stands, as the leaves of the same tree and among trees overlap, which results in a decrease photosynthetic capacity, (2) with increasing stand density, the number and length of lateral branches gradually decreases, especially in the middle and lower parts of a tree, and the shape of the crown changes^38^, and (3) with increasing stand density, branch angles (the angle between the stem of a tree and its main lateral branches) decrease. Alcorn et al*.*^39^ reported that trees with greater branch angles have more open crowns, and greater effective leaf area for photosynthesis.

Surprisingly, the relationship between low-density tree stands and greater tree growth values was not observed in the current study. Trees in the high-density stands at wadi Khoda had greater values for crown depth, crown volume, DBH, tree radius, basal area, and C/H ratio, compared to the trees in the low-density stands at wadi Rahaba (Fig. 5). *Vachellia* trees growing in these two wadis did not exhibit significant differences in crown diameter and tree height. These results suggest that trees growing in these high-density stands were not engaging in intra- and interspecific competition, which often results in lower values for tree growth parameters, as reported in previous studies^37^. Instead, these results suggest that the higher density of trees at wadi Khoda may lead to positive biotic interactions, such as facilitation^7,40^, which would result in the greater tree growth values described here. Alternatively, the greater tree growth values of the *Vachellia* taxa at wadi Khoda may be due to differences in abiotic environmental conditions at the two study sites. For instance, the transect in wadi Khoda had a west to east orientation and the transect in wadi Rahaba had a north to south-east orientation (Fig. 1). Differences in the orientation of these two transects may have resulted in higher soil moisture content at wadi Khoda and led to the greater tree growth values we report. Additional research will be required to determine the relative role of biotic and abiotic factors in the growth of the three keystone *Vachellia* tree taxa in our study area.

The spatial distribution of the *Vachellia* tree taxa in both wadis revealed similar spacing among these trees (Table 2). The mean nearest neighbor distance (r_0_) was slightly shorter for the trees in wadi Khoda, but these differences were not statistically significant (*P* > 0.05). This result occurs because the *Vachellia* tree taxa in both wadis we studied have a scattered distribution, compared the denser stand of trees that typically occur in riparian zones, in which water availability is higher throughout much of the year. This pattern is consistent with that described by Ramsay^41^, concerning the distribution of trees in forests in Darfur, Sudan. The degree to which the observed tree distribution patterns either approach or depart from random expectation (R) were slighter greater than 1.5, indicating the random distribution of individual trees in both wadis^28^. This result occurs because the mean observed distance values (r_0_) were relatively close to the mean expected distance values (r_e_) in both wadis. As pointed out by Cottam and Curtis^27^, this result can be a product of this type of paired nearest-neighbor analysis because many of the nearest neighbor values included in this dataset are duplicates. However, we also believe these results indicate that the distribution of *Vachellia* tree taxa at wadi Khoda and wadi Rahba is not being influenced by human activities.

## Conclusion

The density of *Vachellia* taxa was higher in wadi Khoda, compared to wadi Rahaba. While *V. tortilis* subsp. *raddiana* was the most frequently encountered taxa in both wadis, *V. tortilis* subsp. *tortilis* predominated at lower elevations, with its frequency decreasing with increasing elevation. These results constitute the first step in confirming the keystone status of the three taxa in this region. The next steps in this determination would involve assessing the biotic interactions these taxa engage in with other community members, and their influence on abiotic environmental conditions. The random distribution pattern of the *Vachellia* trees in the two wadis suggests that human activities have had limited effect on their distribution. Management recommendations and practices in Gelba Elba National Park should continue to limit the impact of human activities. Finally, the data reported here serves as a baseline for future assessments of the status of these three keystone tree taxa, in this arid ecosystem.
